# Supplementary material for: Chromothripsis during telomere crisis is independent of NHEJ, and consistent with a replicative origin
Source: Genome Res. 2019 May;29(5):737–49. doi: 10.1101/gr.240705.118 (PMC6499312; doi:10.1101/gr.240705.118)
Supplement: Supplemental Material [file supp_gr.240705.118_Supplemental_file_1.zip › contigs/annotated_contigs/DB57/contig.2.DB57_length_486_mean_cov_6.31275720165.docx]

**DB57_length_486_mean_cov_6.31275720165**

```
TTATGTTTATTGGCTGCTTGTAAGTCTTCTCTTTTGAGAAGTGTCCACTCATGTCCTTTGCTTACTTTTTAATGGGGTTGTTTTTTGAT
 >chr17:67769177-67769331 - E=5e-78
TGTTCAATTGTGTAAGTTCCTTACAGATTTTTAATATCAGACCTTTTTTGGATGCATAGT|TTGCA|CCGCTGCACTCCAGCCTGGGTA
                                                             >chr17:65271405-65271709 -
ACAGAGTGAAACCCTGTCTCAAATAAATACATAAATAAAATAAAATAAGGCTGGGTGCGGTGGCTCACACCTGTAATCCCAGCACTTTG
 E=2e-150
GGGGGCCCAGGCGGGCAGATCACGAGGTCAGGAGTTGGAGACCAACCTGGCCAATATGGTGAAACCCCGTCTCTACTAAAAATATAAAA

ATTTAGCCTGGTGTGGTGGAGCACGCCTGTAATCCCAGCTACTCAGGAGGCTGAGGCAGGAGAATCACTTGAACCTGGGAAGTGGATGT

TGCAGTGAGC|TGAGACCACGCCACTGCACTTCTGCCTGGGTGA
```
